# Update: Interim Guidance for Health Care Providers Caring for Pregnant Women with Possible Zika Virus Exposure — United States (Including U.S. Territories), July 2017

**DOI:** 10.15585/mmwr.mm6629e1

**Published:** 2017-07-28

**Authors:** Titilope Oduyebo, Kara D. Polen, Henry T. Walke, Sarah Reagan-Steiner, Eva Lathrop, Ingrid B. Rabe, Wendi L. Kuhnert-Tallman, Stacey W. Martin, Allison T. Walker, Christopher J. Gregory, Edwin W. Ades, Darin S. Carroll, Maria Rivera, Janice Perez-Padilla, Carolyn Gould, Jeffrey B. Nemhauser, C. Ben Beard, Jennifer L. Harcourt, Laura Viens, Michael Johansson, Sascha R. Ellington, Emily Petersen, Laura A. Smith, Jessica Reichard, Jorge Munoz-Jordan, Michael J. Beach, Dale A. Rose, Ezra Barzilay, Michelle Noonan-Smith, Denise J. Jamieson, Sherif R. Zaki, Lyle R. Petersen, Margaret A. Honein, Dana Meaney-Delman

**Affiliations:** 1 Zika Virus Response Team, CDC.

CDC has updated the interim guidance for U.S. health care providers caring for pregnant women with possible Zika virus exposure in response to 1) declining prevalence of Zika virus disease in the World Health Organization’s Region of the Americas (Americas) and 2) emerging evidence indicating prolonged detection of Zika virus immunoglobulin M (IgM) antibodies. Zika virus cases were first reported in the Americas during 2015–2016; however, the incidence of Zika virus disease has since declined. As the prevalence of Zika virus disease declines, the likelihood of false-positive test results increases. In addition, emerging epidemiologic and laboratory data indicate that, as is the case with other flaviviruses, Zika virus IgM antibodies can persist beyond 12 weeks after infection. Therefore, IgM test results cannot always reliably distinguish between an infection that occurred ***during*** the current pregnancy and one that occurred ***before*** the current pregnancy, particularly for women with possible Zika virus exposure before the current pregnancy. These limitations should be considered when counseling pregnant women about the risks and benefits of testing for Zika virus infection during pregnancy. This updated guidance emphasizes a shared decision-making model for testing and screening pregnant women, one in which patients and providers work together to make decisions about testing and care plans based on patient preferences and values, clinical judgment, and a balanced assessment of risks and expected outcomes.

For these recommendations, **the definition of possible Zika virus exposure has not changed and includes travel to, or residence in an area with risk for mosquito-borne Zika virus transmission or sex with a partner who has traveled to or resides in an area with risk for mosquito-borne Zika virus transmission**. These areas can be found on the CDC “Zika Travel Information” webpage.*

Key recommendations include the following:

**1) All pregnant women in the United States and U.S. territories should be asked about possible Zika virus exposure *before* and *during* the current pregnancy, at every prenatal care visit.** CDC recommends that pregnant women not travel to any area with risk for Zika virus transmission. It is also recommended that pregnant women with a sex partner who has traveled to or lives in an area with risk for Zika virus transmission use condoms or abstain from sex for the duration of the pregnancy.

**2) Pregnant women with recent possible Zika virus exposure and symptoms**^†^ **of Zika virus disease should be tested to diagnose the cause of their symptoms**. The updated recommendations include concurrent Zika virus nucleic acid test (NAT) and serologic testing as soon as possible through 12 weeks after symptom onset.

3) **Asymptomatic pregnant women *with ongoing* possible Zika virus exposure**^§^ **should be offered Zika virus NAT testing three times during pregnancy.** IgM testing is no longer routinely recommended because IgM can persist for months after infection; therefore, IgM results cannot reliably determine whether an infection occurred during the current pregnancy. The optimal timing and frequency of testing of asymptomatic pregnant women with NAT alone is unknown. For pregnant women who have received a diagnosis of ***laboratory*-*confirmed*** Zika virus infection (by either NAT or serology [positive/equivocal Zika virus or dengue virus IgM and Zika virus plaque reduction neutralization test (PRNT) ≥10 and dengue virus PRNT <10 results]) any time before or during the current pregnancy, additional Zika virus testing is not recommended. For pregnant women without a prior laboratory-confirmed diagnosis of Zika virus, NAT testing should be offered at the initiation of prenatal care, and if Zika virus RNA is not detected on clinical specimens, two additional tests should be offered during the course of the pregnancy coinciding with prenatal visits.

**4) Asymptomatic pregnant women who have recent****^¶^** **possible Zika virus exposure (i.e., through travel or sexual exposure) but *without ongoing* possible exposure are not routinely recommended to have Zika virus testing.** Testing should be considered using a shared patient-provider decision-making model, one in which patients and providers work together to make decisions about testing and care plans based on patient preferences and values, clinical judgment, a balanced assessment of risks and expected outcomes, and the jurisdiction’s recommendations. Based on the epidemiology of Zika virus transmission and other epidemiologic considerations (e.g., seasonality), jurisdictions might recommend testing of asymptomatic pregnant women, either for clinical care or as part of Zika virus surveillance. With the decline in the prevalence of Zika virus disease, the updated recommendations for the evaluation and testing of pregnant women with recent possible Zika virus exposure but *without ongoing* possible exposure are now the same for all areas with any risk for Zika virus transmission.

**5) Pregnant women who have recent possible Zika virus exposure and who have a fetus with prenatal ultrasound findings consistent with congenital Zika virus syndrome should receive Zika virus testing to assist in establishing the etiology of the birth defects.** Testing should include both NAT and IgM tests.

**6) The comprehensive approach to testing placental and fetal tissues has been updated.** Testing placental and fetal tissue specimens can be performed for diagnostic purposes in certain scenarios (e.g., women without a diagnosis of laboratory-confirmed Zika virus infection and who have a fetus or infant with possible Zika virus-associated birth defects**). However, testing of placental tissues for Zika virus infection is not routinely recommended for asymptomatic pregnant women who have recent possible Zika virus exposure but *without ongoing* possible exposure and who have a live born infant without evidence of possible Zika virus–associated birth defects.

**7) Zika virus IgM testing as part of preconception counseling to establish baseline IgM results for nonpregnant women *with ongoing* possible Zika virus exposure is not warranted** because Zika virus IgM testing is no longer routinely recommended for asymptomatic pregnant women *with ongoing* possible Zika virus exposure.

CDC continues to evaluate all available evidence and will update recommendations as new information becomes available.

## Zika Virus Infection

Zika virus is a mosquito-borne flavivirus that is closely related to dengue, West Nile, Japanese encephalitis, and yellow fever viruses (*1*). During 2015–2016, Zika virus spread rapidly and caused outbreaks across the Americas; 47 countries and territories in the Americas reported Zika virus outbreaks. However, since early 2017, the reported incidence of Zika virus disease in the region has declined (*2*).

The World Health Organization uses a country classification scheme that describes the epidemiology of Zika virus transmission to aid in geographic risk assessment. Some areas (e.g., American Samoa) have been reclassified to indicate that Zika virus transmission has been interrupted (*3*,*4*), which is reflective of the declining trends in the prevalence of Zika virus disease. As of July 23, 2017, 95 countries and territories have been designated by CDC as areas with any possible risk for Zika virus transmission.

Although the understanding of the consequences of Zika virus infection is improving, diagnosing Zika virus infection accurately continues to present challenges. First, Zika virus is present in body fluids only transiently, which makes confirming the presence of the virus difficult. Second, serologic testing, based on the immunologic response, cannot always reliably determine when infection occurred. Finally, serologic tests are prone to false-positive results and cross-reactivity with other flaviviruses (*5*). With declining prevalence of Zika virus disease (*2*), the probability of false-positive test results increases (*6*). The changing epidemiology further limits the diagnostic capability of currently available Zika virus tests. In this context, CDC has updated the interim guidance for health care providers caring for pregnant women with possible Zika virus exposure to provide new information and highlight current testing limitations.

## Persistence of Zika Virus Nucleic Acid and Immune Response

Data from outbreaks before 2015 indicated that Zika virus RNA was detected in serum for up to 7 days after symptom onset (*1*,*7*). However, in some persons, Zika virus RNA can be detected in body fluids longer than has been documented previously. The Zika Virus Persistence (ZiPer) Study of persons with NAT-confirmed Zika virus disease, recently reported detection of viral RNA in serum 8–15 days after symptom onset in 36% (10 of 28) of participants, 16–30 days after symptom onset in 21% (27 of 129), and >60 days after symptom onset in 4% (three of 79) (*8*). Prolonged detection of Zika virus RNA in serum obtained from pregnant women was also reported; three of the five pregnant women included in the ZiPer study had detectable RNA 46 days after symptom onset, and one had detectable RNA 80 days after symptom onset. This finding is consistent with other small case series (<20 pregnant women in total) that have demonstrated detection of Zika virus RNA for longer than had been previously reported, up to 107 days after symptom onset and 53 days after last exposure (*9*–*15*).

Zika virus IgM antibodies typically become detectable within the first 2 weeks after symptom onset (*1*,*8*,*16*). Published data on the duration of detection of IgM antibodies following Zika virus infection are limited. In the ongoing ZiPer study, IgM antibodies were detected in 34% (17 of 50) of participants at 0–7 days after symptom onset, 100% (28 of 28) at 8–15 days after symptom onset, and 87% (52 of 60) >60 days after symptom onset (*8*). In addition, consistent with what is known about other flaviviruses (*17*), unpublished preliminary data from this study indicate a median of 4 months (122 days, [range = 8–210 days]) to the first negative Zika virus IgM result (*18*). Thus, detection of IgM antibodies might not indicate an infection that occurred during the current pregnancy. Inability to determine the timing of infection through IgM testing is a major challenge for pregnant women and their health care providers, making it difficult for health care providers to counsel pregnant women about the risk for congenital Zika virus infection.

Neutralizing antibodies develop shortly after IgM antibodies and likely persist for many years (*19*). Based on experience with other flaviviruses, previous Zika virus infection is likely to confer prolonged, possibly lifelong, immunity (*20*). Testing is not routinely recommended for pregnant women with a previous diagnosis of ***laboratory*-*confirmed*** Zika virus infection by either NAT or serology (positive/equivocal Zika virus or dengue virus IgM and Zika virus PRNT ≥10 and dengue virus PRNT <10 results). However, in light of the limitations of serologic testing (e.g., cross-reactivity and false-positive test results), for pregnant women without a previous diagnosis of laboratory-confirmed Zika virus infection, including those with laboratory evidence of flavivirus infection or laboratory evidence of presumptive Zika virus or flavivirus infection (Table 1), decisions about testing during a subsequent pregnancy should be made using a shared patient-provider decision-making model. If the decision is made to test, only NAT testing is recommended, because IgM antibody testing might not be able to determine the timing of infection among pregnant women who have had exposure to Zika virus before the current pregnancy.

**TABLE 1:** Interpretation*^,^^†^ of results of nucleic acid and antibody testing^§^^,^^¶^ for suspected Zika virus infection — United States (including U.S. territories), July 2017

Zika virus NAT (serum)**	Zika virus NAT (urine) **	Zika virus IgM^††^	Zika virus PRNT	Dengue virus PRNT	Interpretation and recommendations
Positive	Positive	Any result	Not indicated	Not indicated	**Acute Zika virus infection**
Negative	Positive	Positive	Not indicated	Not indicated	**Acute Zika virus infection**
Negative	Positive	Negative	Not indicated	Not indicated	**Suggests acute Zika virus infection**
					• *Repeat testing on original urine specimen*
					• *If repeat NAT result is positive, interpret as ****evidence of acute Zika virus infection***
					• *If repeat NAT result is negative, repeat Zika virus IgM testing on a serum specimen collected ≥2 weeks after symptom onset or possible exposure or specimen collection date*
					• *If repeat IgM result is positive,^§§^ interpret as ****evidence of acute Zika virus infection***
					• *If repeat IgM result is not positive, interpret as no evidence of Zika virus infection*
Positive	Negative or not performed	Positive	Not indicated	Not indicated	**Acute Zika virus infection**
Positive	Negative or not performed	Negative	Not indicated	Not indicated	**Suggests acute Zika virus infection**
					• *Repeat testing on original serum specimen*
					• *If repeat NAT result is positive, interpret as ****evidence of acute Zika virus infection***
					• *If repeat NAT result is negative, repeat Zika virus IgM testing on a serum specimen collected ≥2 weeks after symptom onset or possible exposure or specimen collection date*
					• *If repeat IgM result is positive,^ §§^ interpret as evidence of acute Zika virus infection*
					• *If repeat IgM antibody result is not positive, interpret as no evidence of Zika virus infection*
Negative	Negative or not performed	Any nonnegative result^¶¶^	≥10	<10	**Zika virus infection; timing of infection cannot be determined**
					• *For persons without prior Zika virus exposure, a positive IgM result represents recent Zika virus infection*
Negative	Negative or not performed	Any nonnegative result^¶¶^	<10	Any result	**No evidence of Zika virus infection**
Negative	Negative or not performed	Any nonnegative result^¶¶^	≥10	≥10	**Flavivirus infection; specific virus cannot be identified; timing of infection cannot be determined**
					• *For persons without prior Zika virus exposure, a positive IgM result represents recent unspecified flavivirus infection*
***For areas where PRNT is not recommended^¶^***					
Negative	Negative or not performed	Positive for Zika virus AND negative for dengue virus	Not performed because PRNT is not recommended		Presumptive Zika virus infection; timing of infection cannot be determined***
Negative	Negative or not performed	Positive for Zika virus AND positive for dengue virus	Not performed because PRNT is not recommended		Presumptive flavivirus infection; specific virus cannot be identified; timing of infection cannot be determined***
Negative	Negative or not performed	Equivocal (either or both assays)	Not performed because PRNT is not recommended		**Insufficient information for interpretation**
					• *Consider repeat testing*
Negative	Negative or not performed	Negative on both assays	Not performed because PRNT is not recommended		**No laboratory evidence of Zika virus infection**

**Abbreviations**: IgM = immunoglobulin M; NAT = nucleic acid test; PRNT = plaque reduction neutralization test.

* Final interpretations of results of Zika virus tests should be performed after all testing is completed.

**^†^** Serology test results that indicate flavivirus infection should be interpreted in the context of circulating flaviviruses.

**^§^** Dengue virus IgM testing is recommended for symptomatic pregnant women as well as for asymptomatic pregnant women residing in areas where PRNT is not recommended.

^¶ ^Currently, PRNT confirmation is not routinely recommended for persons living in Puerto Rico.

** Serum must be submitted for all persons tested for Zika virus infection; a urine specimen for Zika virus NAT testing should always be submitted concurrently with a serum specimen.

**^††^** For laboratory interpretation in the presence of dengue virus IgM results refer to https://www.cdc.gov/dengue/clinicallab/laboratory.html.

**^§§^** **Positive** results include “positive,” “presumptive Zika virus positive,” or “possible Zika virus positive.” These are examples of assay interpretations that might accompany test results; positive serology terminology varies by assay. For explanation of a specific interpretation, refer to the instructions for use for the specific assay performed. Information on each assay can be found at https://www.fda.gov/MedicalDevices/Safety/EmergencySituations/ucm161496.htm#zika under the “Labeling” tab for the specific assay.

^¶¶ ^**Nonnegative** results include “positive,” “equivocal,” “presumptive positive,” or “possible positive.” These are examples of assay interpretations that might accompany test results; nonnegative serology terminology varies by assay. For explanation of a specific interpretation, refer to the instructions for use for the specific assay performed. Information on each assay can be found at https://www.fda.gov/MedicalDevices/Safety/EmergencySituations/ucm161496.htm#zika under the “Labeling” tab for the specific assay.

*** Zika virus IgM positive result is reported as “presumptive positive or flavivirus infection” to denote the need to perform confirmatory PRNT titers against Zika virus, dengue virus, and other flaviviruses to which the person might have been exposed to resolve potential false-positive results that might have been caused by cross-reactivity or nonspecific reactivity. In addition, ambiguous test results (e.g., inconclusive, equivocal, and indeterminate) that are not resolved by retesting also should have PRNT titers performed to rule out a false-positive result. However, PRNT confirmation is currently not routinely recommended for persons living in Puerto Rico.

## Zika Virus Diagnostic Testing

Diagnostic testing for Zika virus infection can be accomplished using molecular and serologic methods; several NAT and serology assays have received Emergency Use Authorization (EUA) from the Food and Drug Administration (FDA) for use on nontissue clinical specimens.^††^^,^^§§^ Zika virus NAT is used to identify viral RNA in clinical or pathologic specimens, and for most persons with suspected Zika virus disease, a positive NAT result confirms acute Zika virus infection. However, despite the high specificity of NAT, false-positive results can occur (*1*,*8*,*16*). In addition, because Zika virus RNA is cleared from blood and other body fluids and tissues, a negative NAT result does not exclude acute Zika virus infection.

Several assays can be used to detect Zika virus IgM antibodies in serum or cerebrospinal fluid. Zika virus IgM tests can be difficult to interpret because of false-positives and cross-reactivity with other flaviviruses, especially in persons who were previously infected with or vaccinated against a related flavivirus (*5*,*21*). Additionally, a negative IgM test result does not rule out Zika virus infection when an IgM test is performed before the development of IgM antibodies or after the antibodies have waned.

PRNT measures virus-specific neutralizing antibody titers and should be performed for Zika and dengue viruses in NAT-negative, IgM-nonnegative (i.e., positive, equivocal, presumptive positive, or possible^¶¶^) specimens (*21*). In primary flavivirus infections (i.e., a person’s first flavivirus infection), PRNT can often identify the infecting virus (*21*). PRNT can also assist in identifying false-positive IgM results. However, PRNT might not discriminate between anti-Zika virus antibodies and cross-reacting antibodies in persons who have been previously infected with or vaccinated against a related flavivirus (i.e., secondary flavivirus infection) (*22*,*23*). In addition, if areas with risk for Zika virus transmission experience increasing levels of dengue virus transmission, the difficulty in differentiating between cross-reactive Zika virus and dengue virus antibodies will further complicate interpretation of test results and diagnosis of Zika virus infection. This is especially concerning at this time, as epidemiologic trends suggest a reduced likelihood of Zika virus transmission in the Americas, compared with 2016 (*2*,*24*).

Efforts to develop and validate Zika virus serologic assays with improved specificity for Zika virus infection and the ability to distinguish a recent infection from a previous infection are ongoing. CDC is currently working with multiple manufacturers to validate tests in development and will update testing recommendations as new information becomes available.

## Updated Interim Guidance for Laboratory Testing of Pregnant Women with Exposure to Areas with Risk for Zika Virus Transmission

**FIGURE 1:**
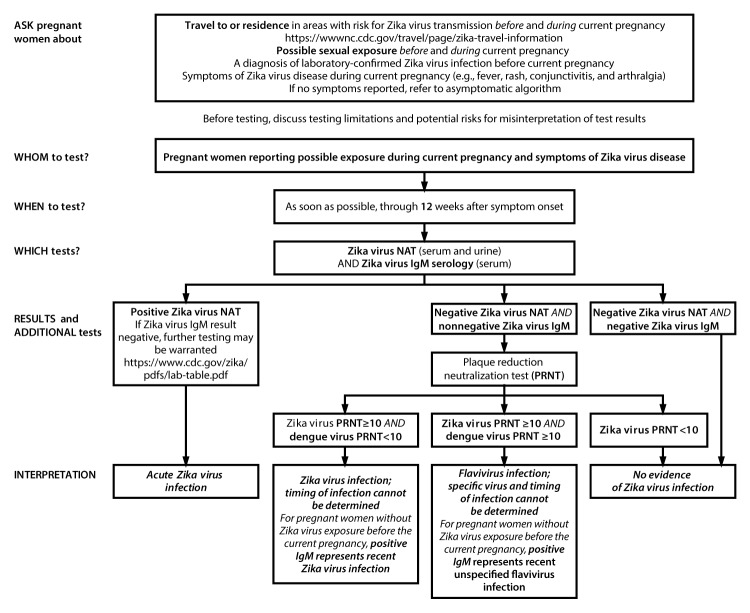
Updated interim testing recommendations*^,^^†^^,^^§^^,^^¶^^,^**^,^^††^^,^^§§^ and interpretation of results**^¶¶^** for symptomatic pregnant women with possible Zika virus exposure***^,^^†††^ — United States (including U.S. territories), July 2017 **Abbreviations**: IgM = immunoglobulin M; NAT = nucleic acid test; PRNT = plaque reduction neutralization test. * Ask about type and duration of Zika virus exposure before and during current pregnancy. Exposure before the current pregnancy might limit interpretation of Zika virus IgM results; pretest counseling can help inform testing decisions. Some patients may choose not to receive Zika virus IgM testing. ^†^ Zika virus testing is not routinely recommended for pregnant women with a previous diagnosis of laboratory-confirmed Zika virus infection by either NAT or serology (positive/equivocal Zika virus or dengue virus IgM and Zika virus PRNT ≥10 and dengue virus PRNT <10 results). ^§^ This algorithm also applies to pregnant women with possible Zika virus exposure who have a fetus with prenatal ultrasound findings consistent with congenital Zika virus syndrome. ^¶^ The duration of detectable Zika virus RNA in pregnant women following infection is not known. Preliminary data suggest that NAT might remain positive for several weeks after symptom onset in some pregnant women. Zika virus IgM antibodies are most likely to be detected within 12 weeks after infection; however, IgM antibodies might be detected for months after infection, limiting the ability to determine whether infection occurred before or during the current pregnancy. ** Dengue virus IgM antibody testing is recommended for symptomatic pregnant women. For laboratory interpretation in the presence of dengue virus IgM results, refer to https://www.cdc.gov/dengue/clinicallab/laboratory.html. ^††^ Nonnegative results include “positive,” “equivocal,” “presumptive positive,” or “possible positive.” These are examples of assay interpretation that might accompany test results; nonnegative serology terminology varies by assay. For explanation of a specific interpretation, refer to the instructions for use for the specific assay performed. Information on each assay can be found at https://www.fda.gov/MedicalDevices/Safety/EmergencySituations/ucm161496.htm#zika under the “Labeling” tab for the specific assay. ^§§^ Currently, PRNT confirmation is not routinely recommended for persons living in Puerto Rico. For laboratory interpretation in the absence of PRNT testing, refer to https://www.cdc.gov/zika/pdfs/lab-table.pdf. ^¶¶^ Despite the high specificity of NAT, false-positive NAT results have been reported. **If both serum and urine specimens are NAT-positive, regardless of IgM antibody results, results should be interpreted as evidence of acute Zika virus infection. If either serum or urine specimen is NAT-positive in conjunction with a positive Zika virus IgM, results should be interpreted as evidence of acute Zika virus infection. **If NAT is only positive on serum or urine and IgM testing is negative, repeat testing on the original NAT-positive specimen. **If repeat NAT is positive, results should be interpreted as evidence of acute Zika virus infection.** If repeat NAT testing is negative, results are indeterminate and health care providers should repeat Zika virus IgM antibody testing on a serum specimen collected ≥2 weeks after symptom onset. **If subsequent IgM antibody test is positive, interpret as evidence of acute Zika virus infection,** but if negative, interpret as no evidence of Zika virus infection. *** Possible Zika virus exposure includes travel to or residence in an area with risk for Zika virus transmission (https://wwwnc.cdc.gov/travel/page/zika-travel-information) during pregnancy or the periconceptional period (8 weeks before conception [6 weeks before the last menstrual period]), or sex without a condom during pregnancy or the periconceptional period, with a partner who traveled to, or resides in an area with risk for Zika virus transmission. ^†††^ For the purposes of this guidance, recent possible Zika virus exposure or Zika virus/flavivirus infection is defined as a possible exposure or infection during the current pregnancy or periconceptional period.

**FIGURE 2:**
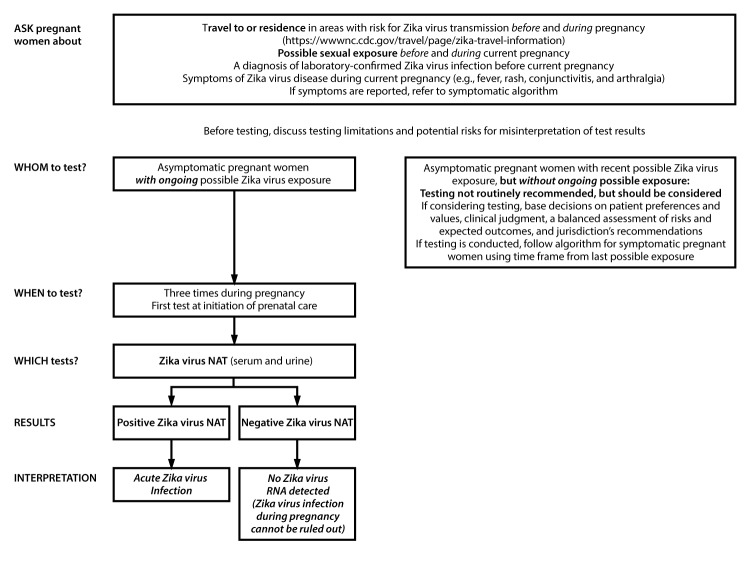
Updated interim testing recommendations*^,^^†^^,^^§^ and interpretation of results^¶^^,^** for asymptomatic pregnant women with possible Zika virus exposure^††^^,^^§§^^,^^¶¶^ — United States (including U.S. territories), July 2017 **Abbreviations**: IgM = immunoglobulin M; NAT = nucleic acid test; PRNT = plaque reduction neutralization test. * Ask about type and duration of Zika virus exposure before and during the current pregnancy. Exposure before the current pregnancy might limit interpretation of Zika virus IgM results; pretest counseling can help inform testing decisions. ^†^ Zika virus testing is not routinely recommended for pregnant women with a previous diagnosis of laboratory-confirmed Zika virus infection by either NAT or serology (positive/equivocal Zika virus or dengue virus IgM and Zika virus PRNT ≥10 and dengue virus PRNT <10 results). ^§^ The interval for Zika virus NAT testing during pregnancy is unknown. Preliminary data suggest that NAT might remain positive for several weeks after infection in some pregnant women. For women without a prior laboratory-confirmed diagnosis of Zika virus, NAT testing should be offered at the initiation of prenatal care, and if Zika virus RNA is not detected on clinical specimens, two additional tests should be offered during the course of the pregnancy coinciding with prenatal visits. The proportion of fetuses and infants with Zika virus–associated birth defects is highest among women with first and early second trimester infections; therefore, conducting all NAT testing during the first and second trimesters might be considered to help identify infections early in pregnancy. However, adverse outcomes have been associated with infection diagnosed in the third trimester; therefore, testing every trimester might be considered. ^¶^ Despite the high specificity of NAT, false-positive NAT results have been reported. **If both serum and urine specimens are NAT-positive, interpretation should be acute Zika virus infection**. If NAT is only positive on serum or urine, testing should be repeated on the original NAT-positive specimen. **If repeat NAT is positive, results should be interpreted as evidence of acute Zika virus infection**. If repeat NAT testing is negative, results are indeterminate and health care providers should perform IgM testing on a specimen collected ≥2 weeks after initial specimen collection. For laboratory interpretation, refer to https://www.cdc.gov/zika/pdfs/lab-table.pdf. ** A negative Zika virus NAT result does not exclude infection during pregnancy because it represents a single point in time. Zika virus RNA levels decline over time, and the duration of the presence of Zika virus RNA in serum and urine following infection varies among pregnant women. Despite Zika virus IgM antibody test limitations (e.g., cross-reactivity with other flaviviruses and prolonged detection for months, presenting challenges in determining the timing of infection), which should be discussed as part of pretest counseling, patients may still choose to receive Zika virus IgM testing. ^††^ Possible Zika virus exposure includes travel to or residence in an area with risk for Zika virus transmission (https://www.cdc.gov/zika/geo/index.html) during pregnancy or the periconceptional period (8 weeks before conception [6 weeks before the last menstrual period]), or sex without a condom, during pregnancy or the periconceptional period, with a partner who traveled to, or resides in an area with risk for Zika virus transmission. ^§§^ Persons *with ongoing* possible Zika virus exposure include those who reside in or frequently travel (e.g., daily or weekly) to an area with risk for Zika virus transmission. ^¶¶^ For the purposes of this guidance, recent possible Zika virus exposure or Zika virus/flavivirus infection is defined as a possible exposure or infection during the current pregnancy or periconceptional period.

As many areas in the Americas move into a subsequent (e.g., a second or third) mosquito season after introduction of Zika virus, testing becomes more complex. Given the evolving situation and the many uncertainties, the updated testing algorithms for symptomatic and asymptomatic pregnant women (Figure 1) (Figure 2) emphasize a shared patient-provider decision-making model. Counseling is recommended before *and* after testing, and Zika virus test results should be interpreted in the context of several limitations (Box). To address new and emerging data, the laboratory interpretations of Zika virus testing (Table 1) have also been updated.

BOX **Key information needed for deciding whether to test and how to interpret serology results**

- Pregnant women with possible Zika virus exposure should be asked about their risk for exposure both *before* and *during* the current pregnancy. Health care providers should ask about the presence of symptoms of Zika virus disease (e.g., fever, rash, arthralgia, and conjunctivitis), and place, duration, and type of travel to assess a woman’s potential for exposure to Zika virus and other flaviviruses (e.g., dengue or West Nile viruses).
- It is important to ascertain whether a woman had exposure to Zika virus before the current pregnancy because Zika virus immunoglobulin M (IgM) antibodies can be detected for months after an infection. A positive Zika virus IgM result could indicate antibodies from infection before the current pregnancy, thus limiting the ability to distinguish between an infection that occurred *before* the current pregnancy and one that occurred *during* the current pregnancy.
- It is important to ascertain whether a woman had exposure to flaviviruses other than Zika virus before the current pregnancy because a positive IgM result might have been caused by cross-reactivity from a previous flavivirus exposure.
- Health care providers and counselors should provide appropriate pretest counseling to inform decisions on whether to test; Zika virus test results should be interpreted within the context of known limitations.
- A negative Zika virus IgM test result, if performed during the recommended time frame, in the setting of a negative Zika virus nucleic acid test (NAT) result, provides some reassurance of absence of Zika virus infection during the current pregnancy. However, a negative Zika virus IgM test result should be interpreted within the context of the limitations of the assay.
- When plaque reduction neutralization testing (PRNT) is indicated and performed during the recommended time frame, a negative PRNT result in the setting of a negative NAT result indicates that there is no laboratory evidence of Zika virus infection.

Health care providers should continue to ask pregnant women at each prenatal visit about possible Zika virus exposure (e.g., travel to, or residence in an area with risk for mosquito-borne Zika virus transmission or sex with a partner who has traveled to or resides in an area with risk for mosquito-borne Zika virus transmission), specifically *before* and *during* the current pregnancy. Health care providers should ask about presence of symptoms of Zika virus disease (e.g., fever, rash, arthralgia, and conjunctivitis) and place, duration, and type of travel to assess a woman’s potential for Zika virus exposure. Data from other mosquito-borne illnesses indicate that intensity of transmission, duration of travel, and type of travel influence the likelihood of infection (*25*,*26*); these factors might also affect the likelihood of Zika virus acquisition. Knowledge of a pregnant woman’s possible exposure to Zika virus *before* and *during* pregnancy is critical contextual information that should be used to tailor pretest and posttest counseling and interpretation of test results (Box). Zika virus IgM test results might be difficult to interpret for pregnant women who have had exposure to any area with risk for Zika virus transmission before the current pregnancy, and this difficulty underscores the importance of shared patient-provider decision-making.

**Pregnant women with recent possible Zika virus exposure and symptoms of Zika virus disease.** Testing for Zika virus infection is still recommended for pregnant women with symptoms of Zika virus disease and possible Zika virus exposure, with the main goal of establishing a diagnosis that accounts for their symptoms, or ruling out Zika virus infection so that an alternative diagnosis can be considered. Negative test results should prompt evaluation for other causes, which might include dengue virus or chikungunya virus infection, depending on the symptoms and epidemiology of circulating viruses.

Concurrent NAT (serum and urine) and serologic testing (serum) is recommended for pregnant women as soon as possible, through 12 weeks after symptom onset (Figure 1). Reports of prolonged detection of Zika virus RNA in symptomatic pregnant women support longer time frames for the performance of molecular diagnostic testing (*8*–*11*,*13*–*15*). However, the proportion of pregnant women with this finding is unknown. Expanding the time frame for NAT testing through 12 weeks after symptom onset allows for a longer period in which to make a NAT-confirmed diagnosis of Zika virus infection in some pregnant women. However, because of the potential for false-positive NAT results (*6*,*27*),*** updated recommendations include NAT testing of both serum and urine and concurrent Zika virus IgM antibody testing to confirm the diagnosis of acute Zika virus infection with more than one test (Table 1).

For women who seek care >12 weeks after symptom onset, Zika virus IgM testing might be considered; however, a negative result does not rule out an infection during pregnancy because IgM levels decline over time. A positive result should be interpreted within the context of the known limitations of serologic testing.

**Asymptomatic pregnant women *with ongoing* possible Zika virus exposure.** For asymptomatic pregnant women *with ongoing* exposure to Zika virus, testing for Zika virus infection should be offered as part of routine obstetric care because it might identify acute infection during pregnancy (Figure 2). Previous guidance recommended IgM testing with reflex NAT once during the first and second trimester of pregnancy for women with *ongoing* possible Zika virus exposure (*28*). IgM testing is no longer routinely recommended because of the limitations of IgM tests and the difficulty in interpreting results.

The optimal timing and frequency for testing asymptomatic pregnant women with NAT alone is unknown; NAT for asymptomatic pregnant women should be informed by jurisdictional trends in Zika virus transmission, the duration of ongoing possible exposure during pregnancy, and data on the duration of Zika virus RNA detection in body fluids. For pregnant women who have received a diagnosis of ***laboratory*-*confirmed*** Zika virus infection any time *before* or *during* the current pregnancy, additional Zika virus testing is not recommended. For women without a prior laboratory-confirmed diagnosis of Zika virus, NAT should be offered at the initiation of prenatal care, and if Zika virus RNA is not detected on clinical specimens, two additional NAT tests should be offered during the course of the pregnancy coinciding with prenatal visits. The proportion of fetuses and infants with Zika virus–associated birth defects is highest among women with first and early second trimester infections (*29*); therefore, conducting all NAT during the first and second trimesters might be considered to help identify infections early in pregnancy. However, adverse outcomes have been associated with infection diagnosed in the third trimester (*28*); therefore testing every trimester might also be considered.

Serologic testing is not routinely recommended for asymptomatic pregnant women with ongoing possible Zika virus exposure because of the potential for prolonged detection of Zika virus IgM, which poses challenges in determining whether the infection and therefore the risk of congenital Zika virus infection, occurred during the current pregnancy. In addition, in areas with ongoing dengue virus transmission, a positive Zika virus IgM result might occur because of serologic cross-reactivity. Despite these limitations, which should be discussed as part of pretest counseling, patients may still choose to receive Zika virus IgM testing (Table 1).

Although a recommendation to consider Zika virus IgM testing as part of preconception counseling to establish baseline IgM results for nonpregnant women *with ongoing* possible Zika virus exposure was previously issued, Zika virus IgM is no longer routinely recommended for asymptomatic pregnant women *with ongoing* possible Zika virus exposure, and therefore baseline preconception testing is not warranted. Zika virus testing is not recommended to determine timing of conception or pregnancy for couples in which one or both partners has had possible Zika virus exposure. Zika virus testing for this purpose is of uncertain value because: 1) IgM testing has diagnostic limitations; 2) Zika virus NAT testing of serum does not reflect persistence in other body fluids (e.g., semen). The current understanding of Zika virus shedding in genital secretions is limited (*30*); testing semen and vaginal fluids for Zika virus is not currently available outside research settings.

**Asymptomatic pregnant women with recent possible Zika virus exposure (i.e., through travel or sex) but *without ongoing* possible exposure**. For asymptomatic pregnant women with recent possible Zika virus exposure (i.e., through travel or sex), but *without ongoing* possible exposure, testing for Zika virus infection is not routinely recommended. However, testing should be considered using a shared decision-making model, one in which patients and providers work together to make decisions about testing and care plans based on patient preferences and values, clinical judgment, a balanced assessment of risks and expected outcomes, and the jurisdiction’s recommendations. Health care providers should consider potential exposure risk factors when deciding whether to advise testing. These include symptoms, type and length of possible exposure, Zika virus transmission trends at location of possible exposure and the use of prevention measures (e.g., insect repellent, appropriate clothing, and condom use). Jurisdictional recommendations may take into account the epidemiology of Zika virus transmission and other epidemiologic considerations (e.g., seasonality and mosquito surveillance and control factors) in areas with risk for Zika virus transmission and, therefore, might include a routine recommendation to test asymptomatic pregnant women either for clinical care or as part of Zika virus infection surveillance.

Although preliminary data indicate that the risk for Zika virus–associated birth defects does not differ by maternal symptom status, testing is not routinely recommended for asymptomatic pregnant women with recent possible Zika virus exposure but *without ongoing* possible exposure to address the increased probability of false positive results in the setting of the declining prevalence of Zika virus disease (*28**,**29*). The limitations of currently available tests and the lack of a vaccine or an effective therapy to prevent congenital infection or mitigate sequelae of Zika virus infection during pregnancy, or in the neonate, underscore the importance of shared patient-provider decision-making. The decision about Zika virus testing should take into account the patient’s unique circumstances and should allow pregnant women to make an informed decision about the utility of testing. If testing is conducted for asymptomatic pregnant women *with recent possible Zika virus exposure, but without ongoing possible exposure*, the testing algorithm for symptomatic pregnant women with possible Zika virus exposure (Figure 1) should be used, applying time frames from last possible Zika virus exposure.

**Pregnant women with possible Zika virus exposure who have a fetus with prenatal ultrasound findings consistent with congenital Zika virus syndrome.** Maternal Zika virus NAT and IgM testing should be performed. Consideration of amniocentesis should be individualized because data about its usefulness in diagnosing congenital Zika virus infection are limited. If amniocentesis is performed as part of clinical care, NAT testing should be performed on amniocentesis specimens. A recent study reported that detection of Zika virus RNA in amniocentesis specimens from pregnancies with a fetus with Zika virus–associated birth defects indicate fetal infection. However, data also suggested that detection of Zika virus RNA in amniotic fluid could be transient and that Zika virus RNA might not always be detectable in amniotic fluid after fetal infection (*13*).

## Updated Interim Guidance for Prenatal Management of Pregnant Women with Laboratory Evidence of Possible Zika Virus Infection^†††^

For pregnant women with laboratory evidence of possible Zika virus infection, serial fetal ultrasounds (every 3–4 weeks) should be considered to assess fetal anatomy, particularly fetal neuroanatomy, and to monitor growth. A study of 17 pregnancies in symptomatic women with laboratory-confirmed Zika virus infection and adverse fetal outcomes in Colombia and a summary of eight published studies of 37 pregnancies reported a median of 18 weeks from symptom onset to prenatal diagnosis of microcephaly (*31*). This finding is consistent with other reports about prenatal diagnosis of microcephaly. Among 37 pregnancies with confirmed or suspected Zika virus infection, a median of 21 weeks (range = 3–29 weeks) from maternal symptom onset to prenatal diagnosis of microcephaly was observed (*31*). Given the length of time for the detection of prenatal microcephaly, prenatal ultrasounds should carefully evaluate the fetal anatomy, particularly the neuroanatomy, to identify brain or structural abnormalities that might occur before microcephaly.

Decisions about performing amniocentesis should be individualized because there is a paucity of data regarding the usefulness of amniocentesis in diagnosing congenital Zika virus infection. The presence of Zika virus RNA in the amniotic fluid might indicate fetal infection; however, a negative result does not exclude congenital Zika virus infection. The optimal time to perform amniocentesis to diagnose congenital Zika virus infection is not known; health care providers should discuss the risks and benefits of amniocentesis with their patients.

This guidance also applies to pregnant women with laboratory evidence of presumptive Zika virus or flavivirus infection; timing of infection cannot be determined (Table 1).

## Updated Interim Guidance for the Evaluation of Placental and Fetal Tissue Specimens for Zika Virus Infection

Detection of Zika virus RNA has been reported in placental tissues and in fetal and infant brain tissue 15–210 days (mean = 81 days) and 119–238 days (mean = 163 days), respectively, from maternal symptom onset (*32*). Among 546 live births with travel-associated possible maternal Zika virus exposure in the 50 U.S. states and the District of Columbia in 2016 for which placental specimens were submitted to CDC, 60 (11%) were positive for Zika virus RNA (*33*). When restricted to live births without a laboratory-confirmed Zika virus infection based on maternal or infant Zika virus testing of serum or urine, 47 of 482 (10%) were positive for Zika virus RNA (*33*). Although, the proportion of live births with positive placental reverse-transcription polymerase chain reaction (RT-PCR) results was relatively low, these results provided definitive evidence of maternal Zika virus infection during that pregnancy. As with serologic and NAT testing of serum and urine, the proportion of pregnancies with a positive Zika virus RT-PCR on tissue specimens is expected to decrease in the setting of declining prevalence of Zika virus disease in the Americas.

Testing placental tissue specimens from pregnancies with possible Zika virus exposure that result in live births can be considered for diagnostic purposes in certain scenarios. It may be considered for symptomatic pregnant women and women with infants with possible Zika virus–associated birth defects, without a definitive diagnosis of laboratory-confirmed Zika virus infection during pregnancy (Table 2). Similar to the updated testing recommendations for asymptomatic pregnant women who have recent possible Zika virus exposure but *without ongoing* possible exposure, testing of placental tissues is not routinely recommended; however, it should be considered for women who have a fetus or infant with possible Zika virus–associated birth defects.

**TABLE 2:** Interim guidance for Zika virus testing* of formalin-fixed, paraffin-embedded placental, fetal, or infant autopsy tissues^†^ for completed pregnancies with possible Zika virus exposure^§^ during pregnancy^¶^ — United States (including U.S. territories), July 2017

Pregnancy outcome	Maternal Zika virus test results on nontissue clinical specimens (e.g., serum, urine)				
	Acute Zika virus infection**	Zika virus infection; timing of infection cannot be determined^††^	Flavivirus infection; timing of infection cannot be determined	>12 weeks after symptom onset or exposure,^§§^ with either negative maternal Zika virus IgM, or no maternal testing conducted	No evidence of Zika virus infection^¶¶^
***Testing of placental tissues***					
Live birth, possible Zika virus–associated birth defects***	Not indicated^†††^	Should be considered to aid in maternal diagnosis			Not indicated^†††^
**Live birth, no obvious Zika virus–associated birth defects at birth**	Not indicated	May be considered to aid in maternal diagnosis on a case-by-case and jurisdictional basis. Not routinely recommended for asymptomatic women with possible Zika virus exposure but *without ongoing* possible exposure			Not indicated
***Testing of placental and fetal tissues***					
**Pregnancy loss, possible Zika virus–associated birth defects**	May be considered to aid in fetal diagnosis	May be considered to aid in fetal and maternal diagnosis			Not indicated^†††^
**Pregnancy loss, no obvious Zika virus–associated birth defects**	May be considered to aid in fetal diagnosis	May be considered to aid in fetal and maternal diagnosis			Not indicated^†††^
***Testing of placental and infant autopsy tissues***					
**Infant death following live birth**	Should be considered to aid in infant diagnosis	Should be considered to aid in infant and maternal diagnosis			Not indicated^†††^

**Abbreviations**: IHC = immunohistochemistry; NAT = nucleic acid test; RT-PCR = reverse-transcription polymerase chain reaction.

* Zika virus testing on formalin-fixed, paraffin embedded tissue specimens is conducted at CDC’s Infectious Diseases Pathology Branch (IDPB) and includes Zika virus RT-PCR on placental and fetal/infant tissues. Zika virus IHC may be performed on placental tissues into the second trimester, fetal tissues from any gestational age, and infant autopsy tissues.

^†^ Placental tissues include placental disc, umbilical cord, and fetal membranes. Zika virus RNA can be focal within placental tissues, and testing of three sections of placenta, one section of umbilical cord, and one section of fetal membrane is recommended (https://www.cdc.gov/zika/laboratories/test-specimens-tissues.html). For pregnancy losses and infant deaths, submission of placental tissues in addition to fetal or infant autopsy tissues, if available, is preferred, but if not available will not preclude placental testing.

^§^ Possible Zika virus exposure includes travel to or residence in an area with risk for Zika virus transmission (https://www.cdc.gov/zika/geo/index.html) during pregnancy or the periconceptional period (8 weeks before conception [6 weeks before the last menstrual period]), or sex without a condom, during pregnancy or the periconceptional period, with a partner who traveled to, or resides in an area with risk for Zika virus transmission.

^¶^ Zika virus testing is not routinely recommended for asymptomatic pregnant women with recent possible Zika virus exposure but *without ongoing* exposure and who have a fetus or infant without Zika virus–associated birth defects.

** In the event of a confirmed maternal acute Zika virus infection or confirmed congenital Zika virus infection in the infant (e.g., a positive NAT), placental testing from live births is not indicated. Currently, placental testing does not routinely provide additional diagnostic information in the setting of a maternal or infant diagnosis of acute or congenital Zika virus infection, respectively.

^††^ For women with no possible Zika virus exposure before the current pregnancy, a positive IgM result likely represents acute Zika virus infection, and placental testing is not indicated.

^§§^ All or part of possible maternal Zika virus exposure, or symptom onset occurred >12 weeks before maternal serum specimen was collected.

^¶¶^ Includes pregnant women with negative Zika virus NAT and negative Zika virus IgM ≤12 weeks after symptom onset or exposure.

*** Possible Zika virus–associated birth defects that meet the CDC surveillance case definition include the following: brain abnormalities and/or microcephaly, intracranial calcifications, ventriculomegaly, neural tube defects and other early brain malformations, eye abnormalities, or other consequences of central nervous system dysfunction including arthrogryposis (joint contractures), congenital hip dysplasia, and congenital deafness (https://www.cdc.gov/zika/geo/pregnancy-outcomes.html). In all cases, infants or fetuses with possible Zika virus–associated birth defects should also be evaluated for other etiologies of congenital anomalies.

^†††^ Testing may be considered on a case-by-case basis, consult CDC for case-specific questions at https://www.cdc.gov/zika/laboratories/test-specimens-tissues.html.

Finally, testing of placental and fetal tissues may be considered in selected scenarios for pregnancies resulting in a miscarriage or fetal loss/stillbirth (and testing of autopsy tissues in the event of an infant death) to provide insight into the potential etiology of the fetal loss or infant death (Table 2), which could inform a woman’s future pregnancy planning. Additional information is available at https://www.cdc.gov/zika/laboratories/test-specimens-tissues.html.

## Implications of Updated Interim Guidance for Laboratory Testing of Pregnant Women with Possible Zika Virus Exposure for the Evaluation and Care of Infants with Possible Congenital Zika Virus Exposure

Interim guidance for the evaluation of infants with congenital Zika virus exposure has been previously published; infants who meet one or more of the published criteria for testing for congenital Zika virus infection should be tested and evaluated in accordance with the updated CDC interim guidance for the evaluation and management of infants with possible Zika virus infection (*34*). However, in light of the updated recommendations that will likely reduce routine Zika virus testing of asymptomatic pregnant women with recent possible Zika virus exposure but *without ongoing *possible exposure, it is critical that pediatric health care providers inquire about possible maternal and congenital Zika virus exposure for every newborn. Infants born to mothers with possible Zika virus exposure during pregnancy but who did not receive testing, including asymptomatic pregnant women with recent possible Zika virus exposure but *without ongoing* possible exposure, should receive a comprehensive physical examination, including standardized measurement of head circumference and newborn hearing screen, as part of routine pediatric care. In addition, based on the level of possible Zika virus exposure (e.g., duration and type of exposure, use of prevention measures, intensity of Zika virus transmission at the location of travel), the provider should consider whether further evaluation of the newborn for possible congenital Zika virus infection is warranted, in which case, a head ultrasound, and ophthalmologic assessment should be considered. Based on results of the evaluation, testing of the infant for Zika virus infection could be considered.

This guidance also applies to infants born to mothers with negative maternal testing in the setting of ongoing possible Zika virus exposure or a possible Zika virus exposure that occurred more than 12 weeks before maternal testing (https://www.cdc.gov/zika/hc-providers/infants-children/evaluation-testing.html). Recommendations for outpatient management during the first 12 months of life include monitoring of head circumference and development and are provided in the updated CDC interim guidance for the evaluation and management of infants with possible Zika virus infection (*34*).

## Prevention of Zika Virus Infection

CDC recommends that pregnant women avoid travel to any area with risk for Zika virus transmission. To prevent Zika virus infection during pregnancy, all pregnant women and their partners should receive counseling on prevention measures including strategies to prevent mosquito bites and sexual transmission of Zika virus (*35*). If pregnant women must travel, CDC recommends strict adherence to strategies to prevent mosquito bites and sexual transmission. Pregnant women living in areas with risk for Zika virus transmission should also follow these strategies. Couples wishing to conceive should receive preconception counseling about how to minimize risks for Zika virus infection (*30*). Other persons at risk for Zika virus exposure should receive information on travel and strategies to prevent mosquito bites and sexual transmission.^§§§^

## Footnotes

*
https://wwwnc.cdc.gov/travel/page/world-map-areas-with-zika .

† Symptoms of Zika virus disease include acute onset of fever, maculopapular rash, arthralgia, or conjunctivitis.

§ Persons *with ongoing* possible Zika virus exposure include those who reside in or frequently travel (e.g., daily or weekly) to an area with risk for Zika virus transmission.

¶ For the purposes of this guidance, recent possible Zika virus exposure or Zika virus/flavivirus infection is defined as a possible exposure or infection during the current pregnancy or periconceptional period (i.e., 8 weeks before conception or 6 weeks before the last menstrual period).

** Possible Zika virus–associated birth defects that meet the CDC surveillance case definition include the following: brain abnormalities and/or microcephaly, intracranial calcifications, ventriculomegaly, neural tube defects and other early brain malformations, eye abnormalities, or other consequences of central nervous system dysfunction including arthrogryposis (joint contractures), congenital hip dysplasia, and congenital deafness) (https://www.cdc.gov/zika/geo/pregnancy-outcomes.html). In all cases, infants or fetuses with possible Zika virus–associated birth defects should also be evaluated for other etiologies of congenital anomalies.

††
https://www.fda.gov/MedicalDevices/Safety/EmergencySituations/ucm161496.htm#zika .

§§
https://www.cdc.gov/zika/laboratories/lab-guidance.html .

¶¶ Terms listed here are only examples of assay interpretation terminology because nonnegative serology terminology varies by assay. For explanation of a specific interpretation, refer to the instructions for use for the specific assay performed. Information on each assay can be found at https://www.fda.gov/MedicalDevices/Safety/EmergencySituations/ucm161496.htm#zika under the “Labeling” tab for the specific assay.

*** Page 52 at https://www.fda.gov/downloads/MedicalDevices/Safety/EmergencySituations/UCM491592.pdf.

††† Laboratory evidence of possible Zika virus infection during pregnancy is defined as 1) Zika virus infection detected by a Zika virus RNA nucleic acid test (NAT) on any maternal, placental, or fetal specimen (referred to as NAT-confirmed) or 2) diagnosis of Zika virus infection, timing of infection cannot be determined or unspecified flavivirus infection, timing of infection cannot be determined by serologic tests on a maternal specimen (i.e., positive/equivocal Zika virus immunoglobulin M [IgM] and Zika virus plaque reduction neutralization test [PRNT] titer ≥10, regardless of dengue virus PRNT value; or negative Zika virus IgM, and positive or equivocal dengue virus IgM, and Zika virus PRNT titer ≥10, regardless of dengue virus PRNT titer). The use of PRNT for confirmation of Zika virus infection, including in pregnant women and infants, is not routinely recommended in Puerto Rico (https://www.cdc.gov/zika/laboratories/lab-guidance.html).

§§§
https://www.cdc.gov/zika/prevention/index.html .
